# A Real-Time Thermal Monitoring System Intended for Embedded Sensors Interfaces

**DOI:** 10.3390/s20195657

**Published:** 2020-10-03

**Authors:** Ouafaa Ettahri, Aziz Oukaira, Mohamed Ali, Ahmad Hassan, Morteza Nabavi, Yvon Savaria, Ahmed Lakhssassi

**Affiliations:** 1 Department of Engineering Computer Science, University of Québec in Outaouais, Gatineau, QC 18X 3X7, Canada; etto01@uqo.ca (O.E.); ahmed.lakhssassi@uqo.ca (A.L.); 2 Electrical Engineering Department, Polytechnique Montréal, Montréal, QC H3T 1J4, Canada; mohamed.ali@polymtl.ca (M.A.); ahmad.hassan@polymtl.ca (A.H.); morteza.nabavi@polymtl.ca (M.N.); yvon.savaria@polymtl.ca (Y.S.); 3 Microelectronics Department, Electronics Research Institute, Cairo 12622, Egypt

**Keywords:** thermal monitoring, industrial embedded applications, sensor interface, heat sources, FRDM-KL26Z, IC, NISA tool, FPGA

## Abstract

This paper proposes a real-time thermal monitoring method using embedded integrated sensor interfaces dedicated to industrial integrated system applications. Industrial sensor interfaces are complex systems that involve analog and mixed signals, where several parameters can influence their performance. These include the presence of heat sources near sensitive integrated circuits, and various heat transfer phenomena need to be considered. This creates a need for real-time thermal monitoring and management. Indeed, the control of transient temperature gradients or temperature differential variations as well as the prediction of possible induced thermal shocks and stress at early design phases of advanced integrated circuits and systems are essential. This paper addresses the growing requirements of microelectronics applications in several areas that experience fast variations in high-power density and thermal gradient differences caused by the implementation of different systems on the same chip, such as the new-generation 5G circuits. To mitigate adverse thermal effects, a real-time prediction algorithm is proposed and validated using the MCUXpresso tool applied to a Freescale embedded sensor board to monitor and predict its temperature profile in real time by programming the embedded sensor into the FRDM-KL26Z board. Based on discrete temperature measurements, the embedded system is used to predict, in advance, overheating situations in the embedded integrated circuit (IC). These results confirm the peak detection capability of the proposed algorithm that satisfactorily predicts thermal peaks in the FRDM-KL26Z board as modeled with a finite element thermal analysis tool (the Numerical Integrated elements for System Analysis (NISA) tool), to gauge the level of local thermomechanical stresses that may be induced. In this paper, the FPGA implementation and comparison measurements are also presented. This work provides a solution to the thermal stresses and local system overheating that have been a major concern for integrated sensor interface designers when designing integrated circuits in various high-performance technologies or harsh environments.

## 1. Introduction

The evolution of integrated circuits has led to the design of increasingly dense circuits that allow much more complex systems to be implemented on smaller surfaces. This high density in SoC (System on Chip) leads to a higher density of power dissipation and overheating problems that can cause disastrous thermal peaks for the circuit.

Thermal detection and monitoring by capacitive integrated interface sensors is, today, one of the most frequent sensors adopted in integrated electronic systems because of its relative simplicity of implementation, high sensitivity, high resolution, low temperature sensitivity and low noise [1]. Capacitive integrated sensors of this kind are widely used in many applications such as gyroscopes [2,3], accelerometers [3,4,5,6], humidity sensors [7,8], displacement sensors [9] and biological sensors [10,11]. As capacitive sensors can be designed to avoid static energy consumption, they are also very suitable for low-power and energy-limited applications, such as battery-operated systems, and wireless sensor arrays designed for the new 5G generation.

**Figure 1:**
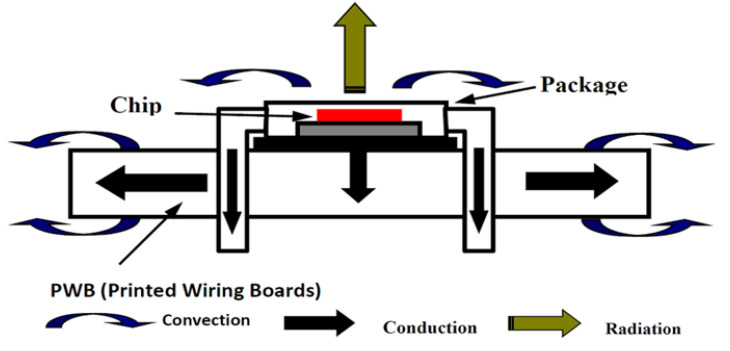
Heat transfer modes in a Systems in Package (SiP).

Systems on Chip (SoC) are widely used in the electronic industry, where complex systems can be integrated on a single chip [12,13,14]. These SoCs can be leveraged to build heterogeneous Systems in Package (SiPs) to create embedded systems with more functionalities. This allows building of high-performance systems that are more compact and that consume less power. Consequently, the high integration density enabled by SoCs increases the power density. This is significant, as it was found that 50% of electronic device failures can be related to high on-chip temperatures [15]. As integrated circuit complexity grows, and as they are composed of various types of modules (memory, processors, vector engines and analog interfaces), it gets more complicated to manage the thermal profile of complex designs [16,17,18,19]. Thus, thermal aspects of SoCs and SiPs need careful consideration. Dealing with issues associated with thermal effects is generally known as thermal management [20]. Junction overheating is a significant issue in complex high-performance electronic systems assembled from miniaturized components. Figure 1 shows the various heat transfer modes in integrated circuits (ICs).

The motivation behind this work is to provide a peak temperature detection approach related to possible thermal management issues in complex electronic systems, such as integrated sensor interfaces used in many industrial applications [21].

The reported study in this paper is not only intended for researchers who are interested in thermal management, but also for designers and manufacturers of ICs who must characterize the thermal dynamics of their products in order to optimize them [22]. Predicting the thermal behavior of ICs and SoCs is motivated by the need of mapping their thermal dynamics. Several methods have been proposed to predict thermal peaks in the literature. For instance, the method used in [21] consists of designing a thermal sensor outside the circuit to control it thermally. This method is no longer practical as circuits get smaller and lighter.

In [22,23], a method for dynamically inserting, operating, and eliminating thermal sensors of FPGA-based systems is proposed. In this paper, we present an integrated peak detection algorithm for thermal monitoring of silicon arrays. The proposed method is used to model an embedded development of sensor board. The novelty of the proposed method is to measure the thermal threshold of any electronic board intended for embedded applications. The presented thermal characterization method of the map is based on two numerical techniques: computational fluid dynamics (CFD) and heat transfer analysis (HTA) [24,25,26].

The rest of the paper is organized as follows. In Section 2, we will review the different relevant materials, parameters and methods used to provide thermal characterization and analysis for a sensor interface under study, as well as the location of the sensor on the FRDM-KL26 board and the operating principle of this type of temperature sensor.

In Section 3, we will present the simulation results found and produced by the Numerical Integrated elements for System Analysis (NISA) tool. These results will be the basis that will guide us in the development of a new algorithm for thermal monitoring proposed in Section 4.

We end the document with Section 5 that describes the simulation results and the hardware implementation; the FPGA results obtained afterwards will be compared to a temperature prediction by our algorithm developed and implemented on the FRDM-KL26 board, after successful download of the VHDL code we developed on the DE1 5CSEMA5F31C6 Altera Cyclone V architecture.

## 2. Materials and Methods

Heat generated from power dissipated in electronic devices can negatively affect performance. In fact, overheating the materials can cause mechanical damages, corrosion and oxidation, which are irreversible consequences. Hence, thermal analysis is an important aspect that should be considered when designing electronic devices. Thermal analysis is normally done as a preliminary study to a stress analysis, where the temperature is analyzed by a structural finite element model.

In this paper, thermal analysis is needed to understand the thermal behavior of the various parts of a SoC/SiP, and this analysis is performed using a finite element. The CFD technique is used with the HTA method to take advantage of their respective strengths when modeling the heat transfer mechanism. The broad specification of this technique is related to the different systems and electronic and physical properties they have, combined with temporal and spatial aspects, to deal with boundary conditions and temperature distributions in dynamic/transient modes [27,28,29].

The temperature distribution on the surface of a chip can be used to predict thermal peaks at localized positions of IC and associated heat sources. The peak detection algorithm developed in this paper can be validated with real measurements and simulations of the thermal dynamics in ICs. This method will provide a means to predict and prevent overheating of integrated circuits in general and of the considered prototype. It is, therefore, important for the designer of integrated circuits, such as the sensor interface studied in this work, to always clearly identify the modes of heat transfer first and to provide, after a study of course, the average rate of heat dissipation to avoid damaging the various electronic components of the board. The heat transfer phenomenon considered in this study is thermal conduction, which is the most appropriate for modeling integrated circuits [30,31,32,33,34].

### 2.1. The FRDM-KL26Z Freescale Board

The FRDM-KL26Z board is an embedded software development platform for the MKL26Z128VLH4 microcontroller. It is part of the Freescale Freedom family of platforms developed by Freescale Semiconductor.

The FRDM-KL26Z platform is built around an ARM Cortex M0+ microcontroller, 64-bit, 48 MHz, 128 kB of Flash program memory, 32 kB of RAM, a USB “Full speed” controller, and digital and analog communication ports. This board also features an RGB LED, 3-axis accelerometer, 3-axis magnetometer, ambient light sensor and capacitive touch slider.

**Figure 2:**
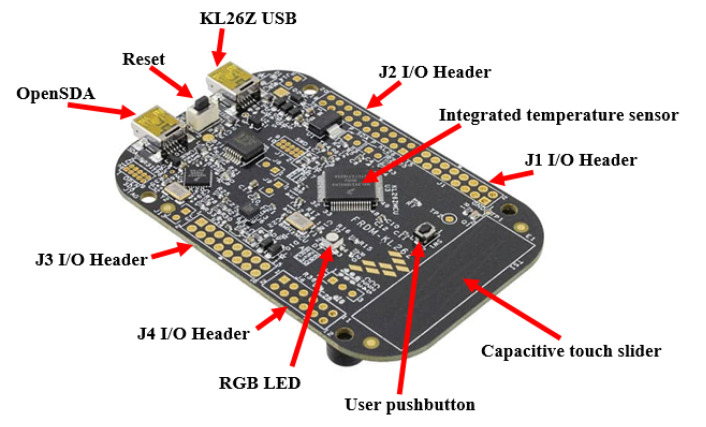
Freescale FRDM-KL26Z board (hardware implementation).

The board is shield compatible for Arduino™ R3. Finally, the board implements the OpenSDA standard for debugging embedded code, which is of interest to all researchers developing interface sensors in the broadest sense. Figure 2 shows the Freescale FRDM-KL26Z board.

Figure 2 shows the location of the temperature sensor and other components of the FRDM-KL26 board. This system allows capturing thermal transients. Then, to control the peak temperature excursions, we have developed a new algorithm in embedded C for real-time thermal management and monitoring using the integrated sensor. Using the MCUXpresso software, we developed a program to activate one or more of the available red or green LEDs depending on the temperature predicted for the Freescale FRDM-KL26Z board.

**Figure 3:**
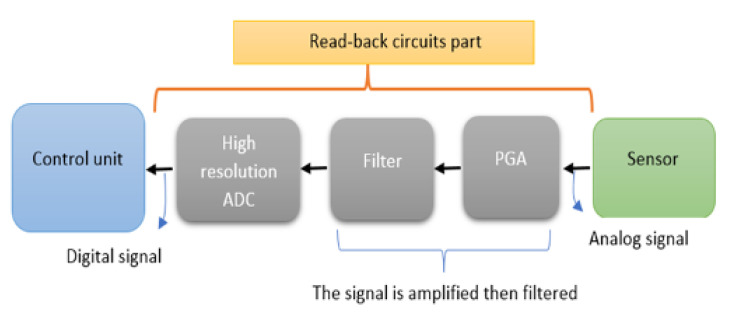
Freescale FRDM-KL26Z board (block diagram).

Figure 3 illustrates the block diagram of the FRDM-KL26Z board in its hardware implementation. It has the following features: ARM Cortex M0+ microcontroller 64 bits, 48 MHz, 128 kB flash memory, 32 kB RAM, USB controller and digital and analog communication ports.

The analog signal provided by the sensor is applied to a programmable gain amplifier, filtered and then digitized by means of a high-resolution analog-to-digital converter (ADC). Then, the generated digital signal is applied to a control unit.

### 2.2. Numerical Heat Transfer Analysis

**Figure 4:**
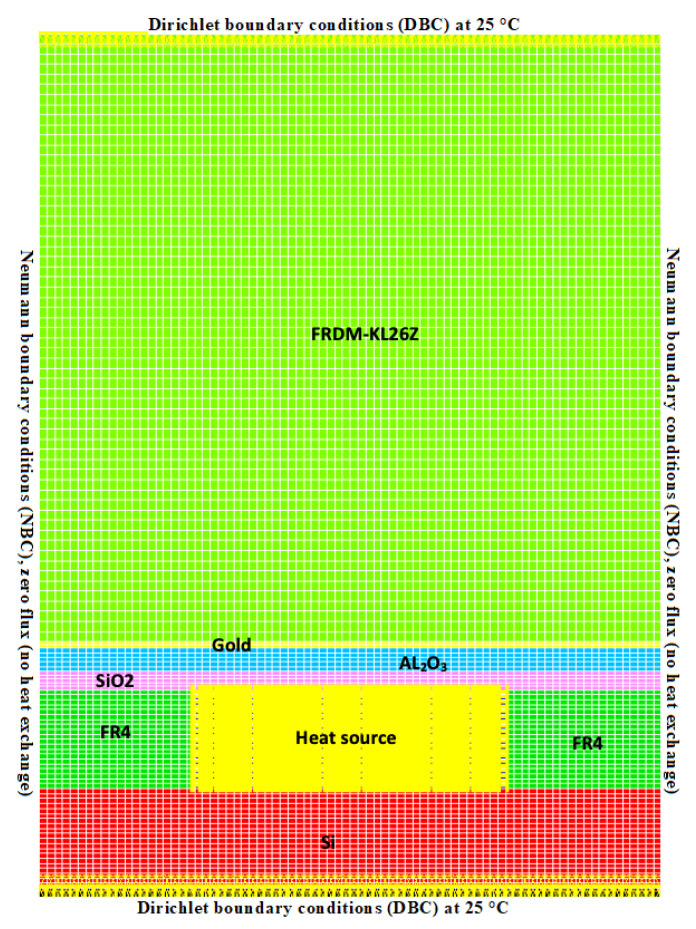
Modeling of the FRDM-KL26Z board with the different materials of which it is composed under the Numerical Integrated elements for System Analysis (NISA) tool.

In this step, a finite element program to predict the thermal behavior of the FRDM-KL26Z board in 2D is used. A wide variety of thermal boundary conditions can be applied using the NISA tool. However, the boundary conditions around the FRDM board cause a problem for the simulations. The simplest approach is to set a constant temperature around the board structure representing the ambient temperature of typically 25 ${}^{{}^{\circ}}$C (298.15 ${}^{{}^{\circ}}$K). In order to solve the thermal diffusion equations in stationary mode, the boundary conditions must be defined beforehand. For the construction of a 2D thermal model of the FRDM, the model is designed to simulate the stationary mode, following predefined steps to build the mechanical structure, the mesh and the boundary conditions. Dirichlet Boundary Conditions (DBC) at 25 ${}^{{}^{\circ}}$C (298.15 ${}^{{}^{\circ}}$K) were applied around the thermal model, and Figure 4 shows the prototype and the 2D FRDM-KL26Z board.

Table 1 below shows the different materials that make up the FRDM-KL26Z board. However, each material has a different thermal conductivity; therefore, this part is essential before the thermal model can be simulated under the NISA tool.

In the transient regime, the following heat conduction Equation (1) or energy conservation law is obtained by considering the equilibrium of the heat flow inside the body of the board used in this paper:

(1) $$\frac{\partial T}{\partial x}\left(k_{x}\frac{\partial T}{\partial x}\right)+\frac{\partial T}{\partial y}\left(k_{y}\frac{\partial T}{\partial y}\right)+\frac{\partial T}{\partial z}\left(k_{z}\frac{\partial T}{\partial z}\right)+P\left(t\right)=\rho C_{p}\frac{\partial T}{\partial t}$$

where kx, ky and kz are the *x*, *y* and *z* thermal conductivity, P(t) is the rate at which heat is generated per unit volume, *t* is time, ρ is the mass density of the material and $C_{p}$ is the specific heat. Table 1 shows the materials used for thermal modeling. Before starting the simulations for the structure representing a room constant temperature, boundary conditions must be defined to perform a transient analysis of the thermal diffusion equations.

Thermal analysis is an important aspect in the design of electronic devices. When materials are heated, they expand and can cause stress that can render the device unusable. In addition, if a part overheats, it may approach the levels causing corrosion and oxidation of the materials. If the part becomes too overheated, it can lose its mechanical and electrical properties. Simplified thermal analysis is normally done as a preliminary study to a stress survey where the temperature is analyzed by a structural finite element model. In the case of this paper, thermal analysis is necessary to achieve a thorough understanding of the different parts of the FRDM-KL26Z board under study. The thermal analysis with finite element models includes the following parts:

1. thermal analysis of the board surfaces,
2. analysis of the convection around the board, and
3. transient thermal analysis.

The execution steps followed during the thermal finite element analysis are represented in the flowchart in Figure 1. Thus, after determining the geometry and heat transfer mechanisms, we model the FRDM-KL26Z board with an appropriate 2D mesh applicable to in-plane thermal problems. In the case of the FRDM-KL26Z board, heat will spread in the plane of the board, and an isothermal profile is needed to perform a thermomechanical stress preliminary evaluation.

## 3. NISA Simulation Results

### 3.1. Simulation of the FRDM-KL26Z Board with the NISA Tool

In this part, the NISA finite element program is used to predict the thermal behavior of the FRDM-KL26Z board in order to obtain the thermal results. A wide variety of thermal boundary conditions can be applied using NISA. Thermal boundary conditions are one of the major problems for the simulation of thermal phenomena in a Cl, and they depend on

1. the method of cooling,
2. the position of the dissipated power and the influence of their surroundings, and
3. the thermal conductivity of the materials in the Printed Circuit Board (PCB).

In this study, we use the finite element program NISA (Numerical Integrated elements for System Analysis) to predict the behavior of the system. A variety of thermal boundary conditions were applied using NISA finite element program. The key information is provided in Table 1, including the boundary conditions, material properties (thermal conductivity, thermal capacity and density), number and types of mesh (triangular) and the boundary conditions. To solve thermal equations, boundary conditions must be defined. Since the FRDM-KL26Z board is relatively thin, the heat flows mainly upward (by natural convection), so the boundary conditions in both horizontal directions in the plane extremities can be modeled by adiabatic-type conditions (zero flux no heat exchange).

**Figure 5:**
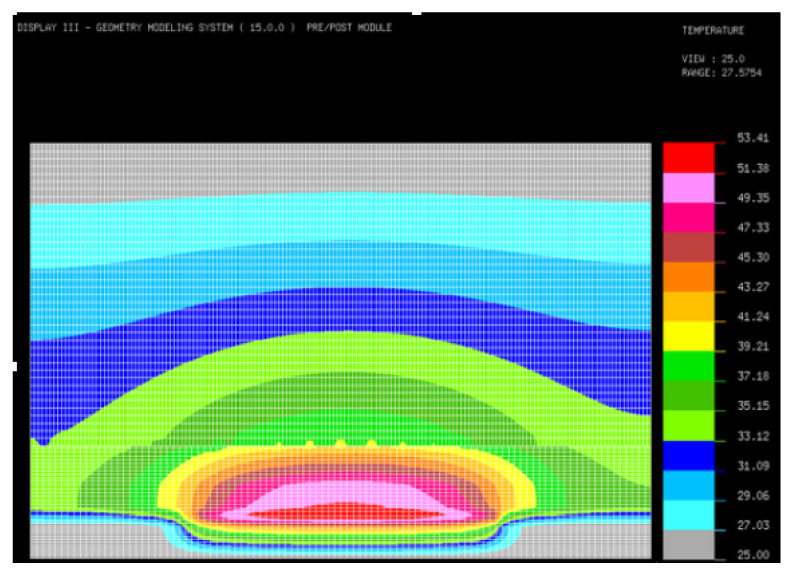
Thermal evolution without convection of the FRDM-KL26Z board as modeled with NISA.

The physical behavior of the map is described by means of partial differential Equation (1) and boundary conditions. The finite element method transforms the partial derivative equations into algebraic equations. This method allows temperature analysis to be performed at different points. However, the vertical boundary condition still causes a big problem for numerical simulations. The simplest approach is to set a constant temperature below the structure at 25 ${}^{{}^{\circ}}$C representing the ambient temperature, and this produces a thermal short circuit. In order to solve thermal equations, the boundary conditions must be defined. Figure 5 shows the first simulation result of the heat sources on the FRD-KL26Z board.

The first simulation of our map under the NISA tool gives a good idea of the thermal behaviour and diffusion of the heat sources in FRDM-KL26Z, and it also shows the temperature increase in an unimaginable way at 53 ${}^{{}^{\circ}}$C, which gives a good overview of the total power dissipation to be expected throughout the map.

### 3.2. Thermal Management for FRDM-KL26Z by Convection

In order to have good thermal management at the FRDM-KL26Z board, it is necessary to control the temperature and its gradient from the first step of the design. In this phase, our goal is to ensure thermal management for the whole board in order to use the interface sensor correctly by simulating a simple 2D thermal model. However, the heat source simulations are needed for the map in order to establish its thermal mapping. This leads to making simulations, which allows us to understand the thermal effect on the map, before use in sensitive applications, as any thermal overflow leads to a false result. In applications where power dissipation is low, the analysis of natural convection has a lower computational load and is sufficient for this part of the study (our case study in this paper). However, due to the high level of power dissipation in the FRDM-KL26Z board, forced convection analysis is the most efficient way to dissipate the amount of heat produced. Table 2 shows the different convection coefficients associated with different types of analysis [35].

In our analysis, it can be seen that the second type of convection is impossible, as we will need an intervention from outside the map, whereas the first type is more than sufficient to properly evacuate the accumulated thermal energy. However, according to our study under the NISA tool, forced convection can respond appropriately if it is combined with a correct configuration; in this way, it can provide an adequate method to keep the thermal stability of the FRDM-KL26Z board.

### 3.3. Simulation by Natural Convection

**Figure 6:**
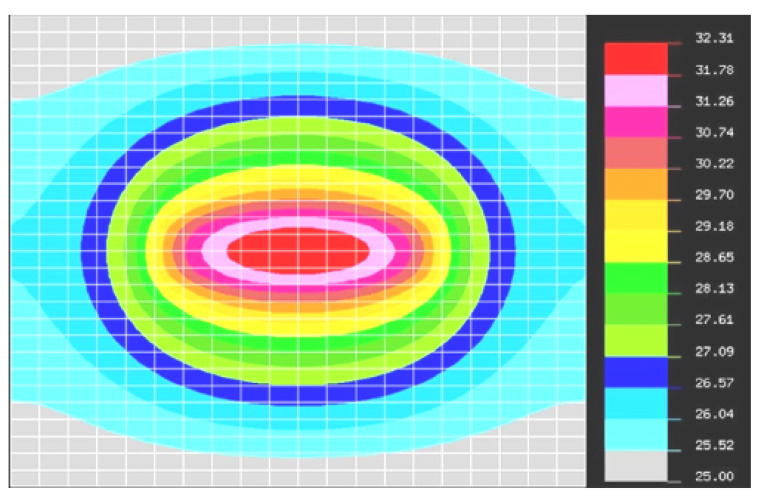
Thermal evolution with natural convection of the FRDM-KL26Z board as modeled with NISA.

In this section, the results of the thermal source simulation of the FRDM-KL26Z board are presented using natural convection. A synthesis is performed to study the thermal behavior of the whole map. We used the heat transfer exchange coefficient h = 5 $W/m^{2}K$ (see Table 2) and then we proceeded to the configuration of boundary conditions for the type of analysis by natural convection. Figure 6 shows the simulation of the FRDM-KL26Z map by natural convection under the NISA tool.

**Figure 7:**
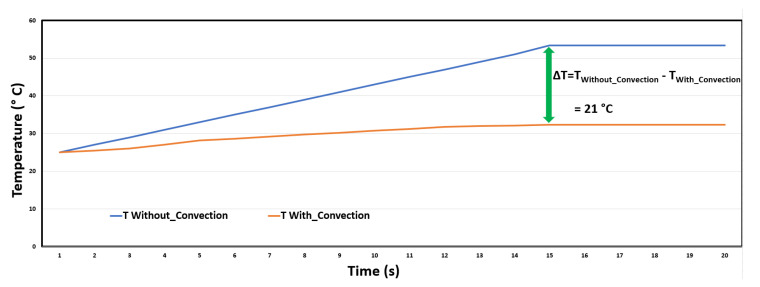
Difference in temperature between analyses without convection and with natural convection.

Figure 6 shows a transient thermal simulation of the FRDM-KL26Z map caused by natural convection as modeled by the NISA tool, which gives a good idea of the thermal evolution of the map. Moreover, we can see that the temperature decreased to 32 ${}^{{}^{\circ}}$C with a difference of 21 ${}^{{}^{\circ}}$C. The characterization of the thermal dynamics of the FRDM-KL26Z board is based on two numerical techniques: CFD (Computational Fluid Dynamics) and the other HTA (Heat Transfer Analysis). The CFD technique, which already takes into account the strength of the heat flow mechanism on the electronic board, and the physical properties will be linked with the temporal and spatial aspects of the HTA to deal with boundary conditions and temperature distribution in the dynamic regime. Thus, the temperature distribution on the surface of the map will be used for the prediction of the thermomechanical stress localized at the sensor location. To visualize the difference between the two types of analyses, a graph (Figure 7) is used to clearly show the actual difference between the two types of analyses using natural convection and non-convection in succession.

We can clearly see the difference between analyses by natural convection and non-convection. We, thus, conclude that natural convection is the best way to reduce the temperature sufficiently in terms of the power dissipated by the FRDM-KL26Z board. Thermal stability from 15 s is also observed. In applications where power dissipation is low, natural convection cooling is economical and easy to implement. However, due to the high level of power dissipation in the ASIC circuit, forced convection cooling is required due to the enormous amount of heat to be removed.

## 4. Development and Execution of the Proposed Algorithm

### 4.1. Thermal Peak Detection Algorithm Applied to the FRDM-KL26Z Board

**Figure 8:**
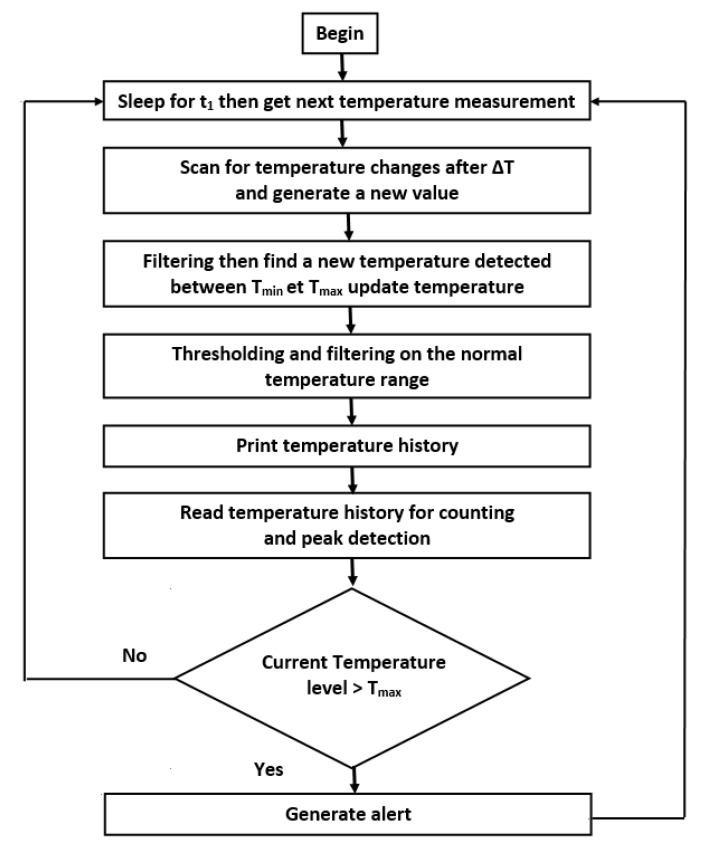
Proposed approach for the detection of thermal peaks applied to the FRDM-KL26Z board.

A real-time temperature prediction algorithm that would allow timely intervention is presented in this paper. The objective of this new methodology presented in Figure 8, which is programmed in C language and executed on the MKL26Z128VLH4 microcontroller using the MCUXpresso software, and it can activate a red or green LED depending on the predicted temperature for the Freescale FRDM-KL26Z board. This is a real-time thermal monitoring algorithm for embedded sensor interfaces.

Figure 8 describes the proposed methodology of thermal peak detection with the FRDM-KL26Z board with a thermal sensor. It is programmed in C language to detect the thermal peak and give an alarm. The predictor was developed using the embedded software development platform for the FRDM-KL26Z board, which is part of the Freescale Freedom platform family.

**Figure 9:**
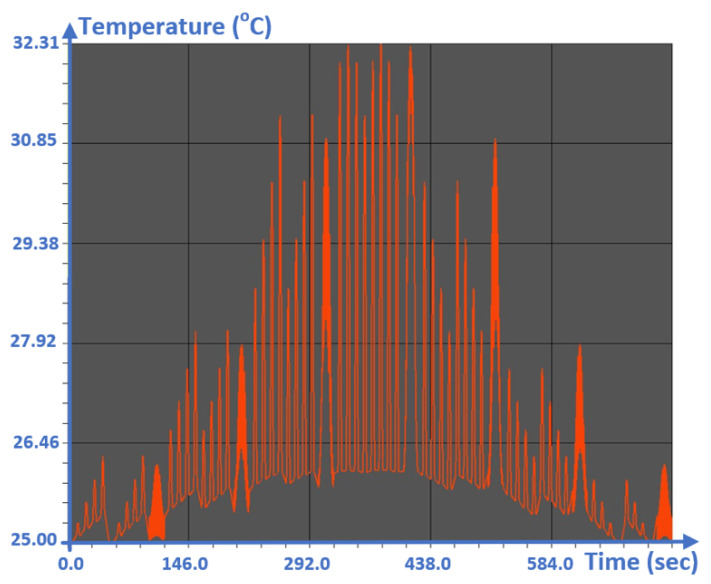
Location of the thermal peaks in real time that are extracted using the NISA numerical analysis tool for the FRDM-KL26Z board.

The results of typical temperature monitoring in the form of real-time thermal peaks were extracted using the NISA numerical analysis tool and presented in Figure 9. The measured value is compared to a peak value, which in this case was 32 ${}^{{}^{\circ}}$C defined in the program before starting the monitoring. The LED lights green if the measured and peak values are equal, provided that the maximum does not exceed 32 ${}^{{}^{\circ}}$C. This condition indicates that the board is cold, and under normal conditions the green LED remains lit. Conversely, the LED turns red if this is not the case.

The program is initialized by generating the current temperature of the card (measured automatically) in order to set the first average between the current temperature value and the maximum temperature value to 32 ${}^{{}^{\circ}}$C. Then it starts to display the different temperature values during the runtime. If it has not yet changed its status, the green LED stays on; if the temperature is above the average (32 ${}^{{}^{\circ}}$C), the red LED lights up to warn that the temperature threshold set in the program has been exceeded. If the temperature is within the average but there has already been a change of state, no LED lights up.

### 4.2. Execution of the Proposed Algorithm

**Figure 10:**
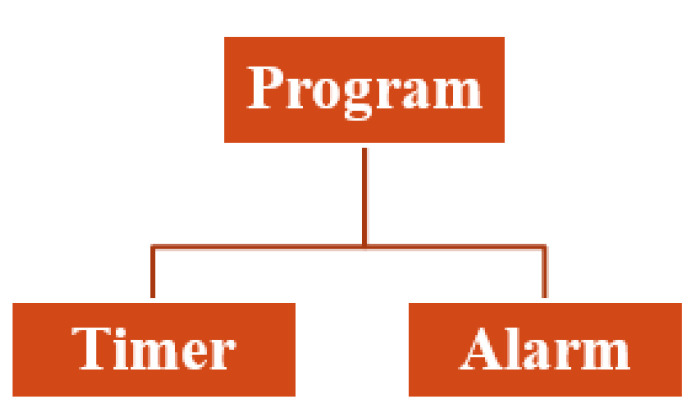
Structure of the embedded C program.

Using the MCUXpresso development platform, a C control and monitoring program was developed and compiled. This program comprises a counter and an alarm. Figure 10 shows the structure of the program.

**Figure 11:**
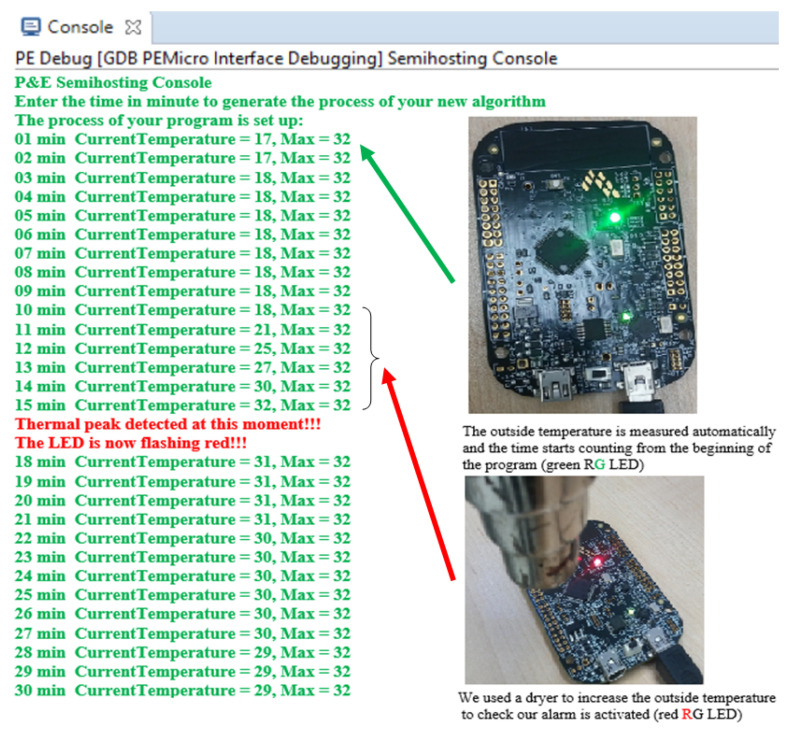
Proposed real-time C code execution console associated with the experimental response of the FRDM-KL26Z board on different states.

The methodology for monitoring and detection of thermal peaks proposed in this document is based on the automatic measurement performed by a temperature sensor integrated in the FRDM-KL26Z board. A platform has been configured to detect thermal peaks in the case of a single heat source by comparing several temperature measurements during transient heating. Figure 11 shows the console of the MCUXpresso tool when predicting the real-time temperature values associated with the experimental response of the FRDM-KL26Z board on different thermal states. The idea presented here is to repeat the process each time a temperature peak or overshoot is exceeded. We used the dryer in order to increase the outside temperature, which will be measured automatically.

The outdoor temperature has a great impact on the measurement results, as we are dealing with a very sensitive sensor integrated in the FRDM-KL26Z board, which is able to detect temperature peaks in real time thanks to an integrated C code (Figure 11). By using the NISA simulator to extract different thermal peaks from the FRDM-KL26Z card in transient (Figure 9), the algorithm works inside the system and operates autonomously. This algorithm has been developed and integrated using the MCU microcontroller (MCU) based on an Arm^®^ Cortex^®^-M0+ core using real-time embedded C. This algorithm is able to generate and detect the current temperature and the maximum temperature value at 32 ${}^{{}^{\circ}}$C (Figure 11), and we can do real-time thermal management around the board. However, we wanted a marking program that did not get stuck when there were potential measurement errors (0.1 ${}^{{}^{\circ}}$C according to the datasheet of the FRDM-KL26Z board) or user input errors. To do this, we simply decided to warn the user that there could be potential measurement errors that did not exceed 0.1 ${}^{{}^{\circ}}$C, knowing that the map displays two options “1” or “2” in the console. Either the user or the code will be able to choose the right option to initialize the operation of the system. To verify and validate the efficiency of our aglorithm, we implemented the FPGA board for rapid prototyping.

## 5. Experimental Implementation FPGA and Results

In this section, we will present the results of FPGA implementation, including theoretical results and thermal simulations performed by our algorithm for the FRDM-KL26Z board at very large scale. The VHDL code and its test bench were implemented in the operation intended to facilitate the development and verification of the algorithm. This architecture will be modeled in high-level language, simulated to evaluate their performance and implemented on the FPGA board, DE1 cyclone V. Our design was divided into three main parts: simulation, synthesis and implementation of the VHDL code. After generating the two files code.vhd (the primary file system) and test-bench.vhd (the test bench) with the Quartus Prime Navigator’s “System Generator”, which synthesizes and generates the Register Transfer Level (RTL) files, we implemented and displayed the maximum temperature on the FPGA.

### 5.1. Creating and Generating RTL Files with Quartus Prime

**Figure 12:**
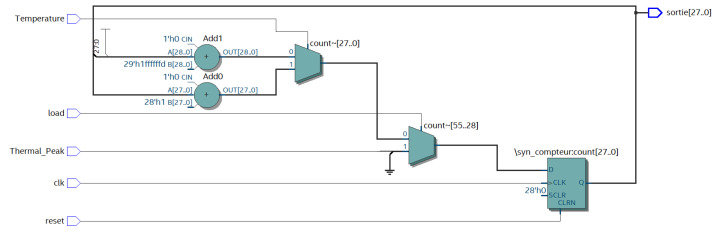
RTL (Register Transfer Level) design of the thermal peak control unit implemented with the Quartus Prime tool.

**Figure 13:**
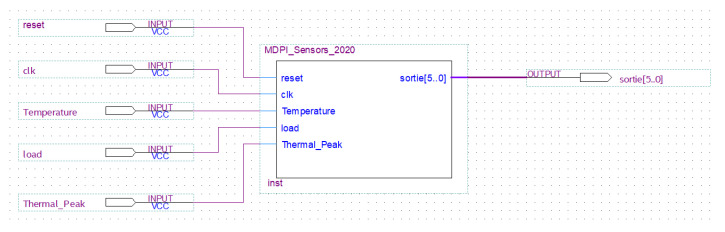
High-level design of the Thermal Peak Control Unit implemented with Quartus Prime.

This part presents the description of the architecture of the thermal peak control unit using a VHDL code editor, with the system generator of the Quartus Prime Navigator, which can synthesize the design and generate the RTL files as shown in both Figure 12 and Figure 13.

### 5.2. Logical Simulation of VHDL Code

**Figure 14:**
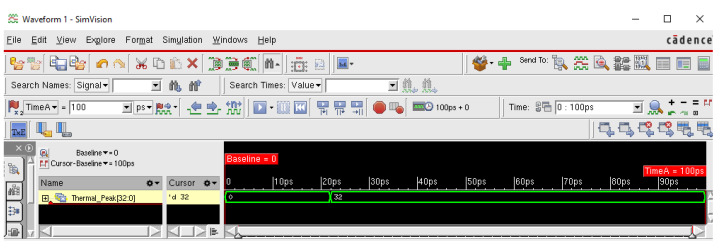
Simulation and detection results of the maximum thermal peak as modeled with the NClaunch Cadence tool.

The main objective of this section is to perform the simulation with Cadence NClaunch tool in order to verify the physical application of our algorithm for any integrated circuit designed according to the standard front end and back end, to validate the thermal analysis with the finite element method (FEM) and to dedicate the maximum temperature value (32 ${}^{{}^{\circ}}$C). Figure 14 shows the correct value of the simulated temperature found.

Figure 14 shows the correct value of the simulated temperature found, i.e., 32 ${}^{{}^{\circ}}$C, which is the correct value displayed by the console (Figure 11) as well as the one extracted by the NISA tool (Figure 9).

### 5.3. Implementation on the FPGA Board

**Figure 15:**
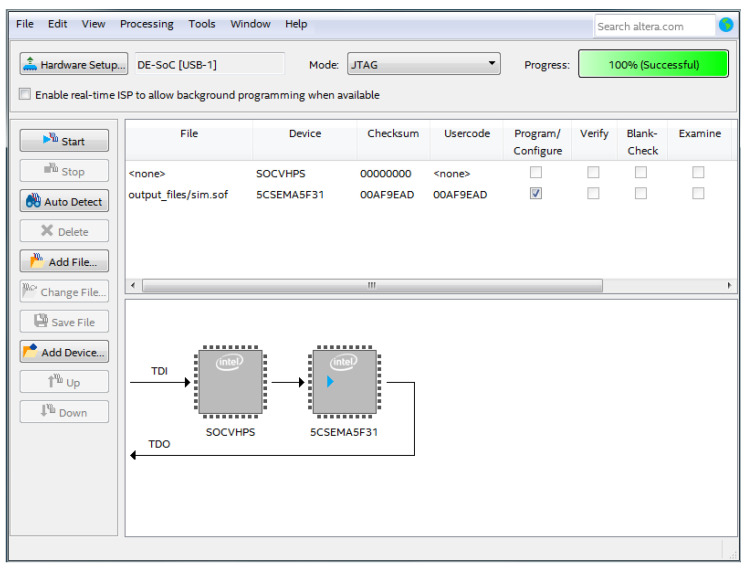
VHDL code of the DE1 Altera Cyclone V architecture.

Once the compilation is completed, after assigning the pins, our program was ready to be downloaded on the DE1 cyclone V board family 5CSEMA5F31C6, and Figure 15 shows that the code was successfully downloaded on the board.

**Figure 16:**
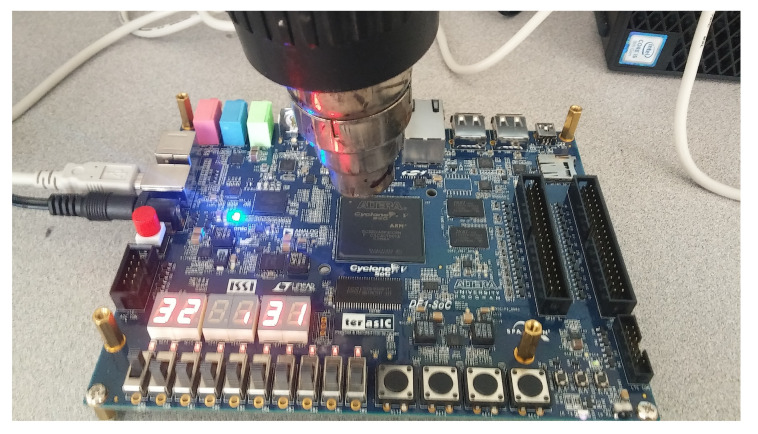
Values of the maximum thermal peak available on the Cyclone V FPGA board family 5CSEMA5F31C6.

After downloading the VHDL code, the program ran and the results were displayed. The clock was set to 50 MHz, so the outputs should change with a frequency of 50 MHz. To raise the temperature on the DE1 FPGA board to the maximum and to check our results obtained with different simulations, a dryer was used to calibrate the sensor on the FPGA board. Figure 16 shows the maximum thermal peak value available on FPGA cyclone V.

After modeling and synthesis, planning, placement and routing tools with the specialized post-synthesis verification tools, integrated in the Quartus Prime tool, were exploited to program our algorithm on the FPGA board. The simulation with Cadence NClaunch tool gave us an overview of the implementation compared to the logical simulation. The results of the logic simulation and the implementation were satisfactory, which validates our algorithm (Figure 8). Moreover, this work can be applied to various complex industrial applications.

## 6. Conclusions

This paper proposes an integrated thermal peak detection algorithm for transient thermal monitoring of electronic boards. The algorithm is based on a discrete thermal measurement validated by the NISA numerical modeling tool. The thermal measurement results are displayed on the console of the MCUXpresso tool, which allows users to detect thermal peaks in the sensor interface. This method has been applied to a Freescale FRDM-KL26Z development board. A real-time software implementation of the algorithm coded with the C language was developed and implemented on the FPGA board to validate the efficiency of the algorithm. The latter can be used to manage the temperature peak occurring during the transient warming mode on the chip before damages are induced by thermal effects. The proposed method allows users to intervene at the right time to mitigate possible adverse thermal effects in embedded sensor interfaces dedicated to electronic industrial applications. In particular, sensor interfaces need thermal stability, and are very sensitive to different thermal effects, in order to deliver the right temperature values. In this paper, we adopted the strategy allowing an alarm value to never be negative; this could avoid some bugs. Hence, when the alarm value was zero, it was deactivated or positive. When the alarm was activated, its value decremented every second. In addition, when an alarm was triggered, the RGB LED flashed for one second. The proposed real-time thermal monitoring system for integrated sensor interfaces is valuable for embedded industrial applications. It can be used to help developers perform real-time and automated thermal monitoring. This work provides a framework for measurement and control of environmental parameters in embedded electronic systems. 

## Figures and Tables

**Table 1:** Material properties used for the thermal simulations.

Material Type	Width (mm)	Depth (mm)	Length (mm)
FR4	6.25	0.3	11.52
$AL_{2}O_{3}$	5.9	0.462	11.6
Gold	5.4	0.038	10.2
Si	5.4	0.13	10.2
$SiO_{2}$	9.66	4.86	13.23

**Table 2:** Convection coefficients associated with different types of analysis.

Cooling Type	Heat Transfer Coefficient ($W/m^{2}K$)	Comments
Air, free convection	3–12	Typically about 5
Air, forced convection	10–100	Typically about 50
